# Urea influences amino acid turnover in bovine cumulus-oocyte complexes, cumulus cells and denuded oocytes, and affects *in vitro* fertilization outcome

**DOI:** 10.1038/s41598-018-30774-2

**Published:** 2018-08-15

**Authors:** Rasoul Kowsar, Vahid Norozian Iranshahi, Nima Sadeghi, Ahmad Riasi, Akio Miyamoto

**Affiliations:** 1 Department of Animal Sciences, College of Agriculture, Isfahan University of Technology, Isfahan, 84156–83111 Iran; 2 Graduate School of Animal and Food Hygiene, Obihiro University of Agriculture and Veterinary Medicine, Obihiro, Hokkaido 080-8555 Japan; 3 FKA, Animal Husbandry and Agriculture Co., Isfahan, Iran

## Abstract

High-protein diets often lead to an increase in urea concentration in follicular fluid of dairy cows, which may reduce oocyte competence. In the present study, maturation media were supplemented with urea (0, 20, 40 mg/dl), and amino acids (AAs) turnover was evaluated in the 24-h spent media of specimens (cell types), bovine cumulus-oocyte complexes (COCs), cumulus cells (CCs), or denuded oocytes (DOs). The main effects of urea and cell type, and their interaction were significant on the individual turnover (expect threonine, glycine, and tyrosine) and total turnover, depletion, and appearance of AAs. The results showed a high level of urea and DOs increased the depletion of all AAs and that of essential and non-AAs, respectively. Sensitivity analysis revealed the highest sensitivity of isoleucine, lysine, and tryptophan to urea, especially in DOs. Principal component analysis (PCA) evaluated the strong correlations between the turnover of: (1) glutamine, aspartic acid or glycine, and developmental competence and fertilization of COCs; (2) serine, isoleucine, valine or glutamic acid, and cleavage rate of DOs; and (3) serine, glutamine, aspartic acid or alanine, and CCs viability. In conclusion, urea significantly changed the turnover of AAs by COCs, CCs and DOs, and reduced the subsequent developmental competence of bovine oocytes.

## Introduction

To support the potential of milk production, high-producing dairy cows are often fed high-protein diets (17 to 19% crude protein^1^) and may, in turn, experience a reduction in fertility^2^. High protein intake may lead dairy cows to suffer from high concentrations of urea in both blood and reproductive fluids, which may lead to reduced fertility^3,4^. Indeed, a high correlation exists between urea concentrations in both plasma and follicular fluid in cows^3^. Iwata *et al*.^3^ found that follicular concentration of urea was around its normal range in serum, 16 mg/dl. They also reported a significant correlation between follicular concentrations of urea and lower developmental competence of oocytes, and suggested BUN as a predictable index for developmental competence of oocytes. Sinclair *et al*.^5^ reported that cytoplasmic and nuclear maturation of oocyte were sensitive to nutrition conditions. They found that oocytes collected from heifers fed high BUN-generating diets had a lower developmental competence. So, high blood urea nitrogen (BUN) may negatively affect oocyte quality and reduce the fertility of healthy dairy cows^5–7^.

Using 18 identified amino acid (AA) transporters, murine oocyte can utilize external AAs^8^. Many researchers suggest the turnover of AAs as a non-invasive marker for evaluating the developmental competence of oocytes and embryos^9–12^. For example, aneuploid embryos negatively showed increased AAs turnover compared to normal embryos^13^. Houghton *et al*.^11^ reported that embryos arrested on day 2–3 post-fertilization exhibited greater AAs turnover compared to those developed to the blastocyst stage. In addition, oocytes having a poor developmental competence showed substantial AAs turnover^9^. Indeed, un-cleaved oocytes may consume (deplete) more nutrients, such as amino acids, to meet their needs for DNA repair^9^.

Importantly, cross-talk exists between oocyte and its surrounding cumulus cells (CCs)^14,15^. The communication between oocyte and CCs is important for the survival of CCs, development of follicles, metabolism, AAs uptake, and competence of oocytes^14–16^. For example, oocytes do not express alanine transporter and need CCs to uptake alanine^17^. To date, the effect of urea on the turnover of amino acids in bovine cumulus-oocyte complexes (COCs), cumulus cells (CCs), or denuded oocytes (DOs) have not yet been addressed.

We, therefore, hypothesized that urea at: (1) 0 mg/dl; (2) 20 mg/dl (equivalent to 9.3 mg/dl BUN found in healthy dairy cows under low protein diets); and (3) 40 mg/dl (equivalent to 18.7 mg/dl BUN found in healthy dairy cows fed high-protein diets^7,18^) may differently change the turnover of AAs in bovine COCs, CCs, and DOs. Moreover, marginal effects and principal component analysis (PCA) were also employed to estimate the sensitivity of amino acids to urea, and to identify the association between amino acid turnover and the viability of cumulus cells or subsequent developmental competence of COCs and DOs.

## Results

### Turnover of essential amino acids

Data were calculated in pmol/h and corrected based on the total number of COCs or DOs within each pooled-sample, according to published calculations^9–11^. Negative values mean that AAs disappeared from the maturation medium (depletion). Positive values mean that AAs appeared at higher concentrations in maturation medium after 24-h incubation (appearance).

**Figure 1:**
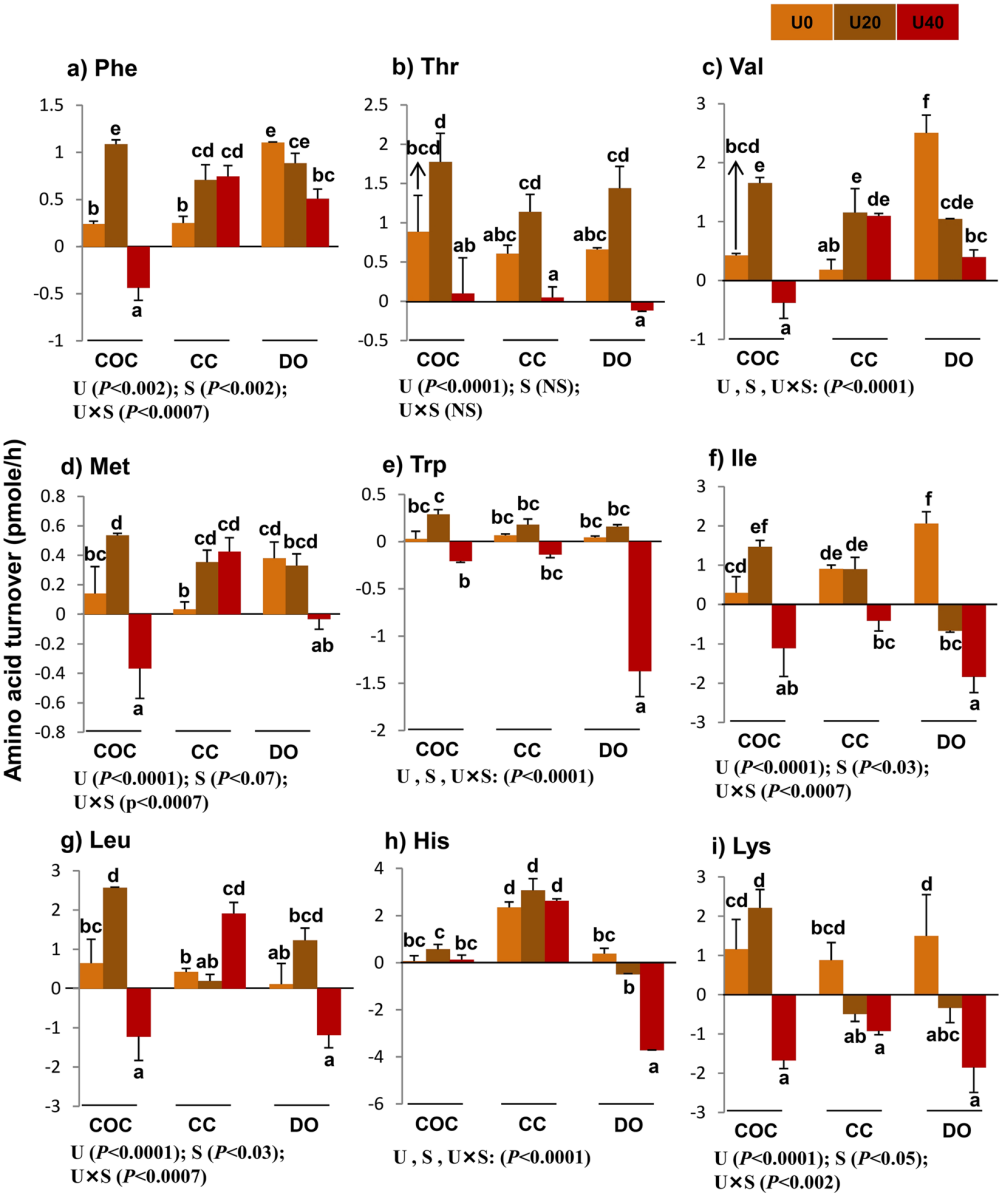
Effect of urea supplementation (0, 20, or 40 mg/dl) in maturation medium on the net depletion/appearance of essential amino acids by bovine cumulus-oocyte complex, oocytectomized cumulus cells, or denuded oocytes after 24-h incubation. U0: control group (without urea); U20: 20 mg/dl urea; U40: 40 mg/dl urea. COC: cumulus oocyte complex; CC: cumulus cells; DO: denuded oocytes; U: the main effect of urea; S: the main effect of specimens (cell type: COCs, DOs, and CCs); U × S: interaction between urea and cell type. Data are presented as least squares means (LSM) ± standard error mean (SEM) indicated by two-way ANOVA over PROC GLM. Different letters (a, b, c, etc.) indicate significant differences between the treatments at *P* < 0.05. NS: non-significant.

**Figure 2:**
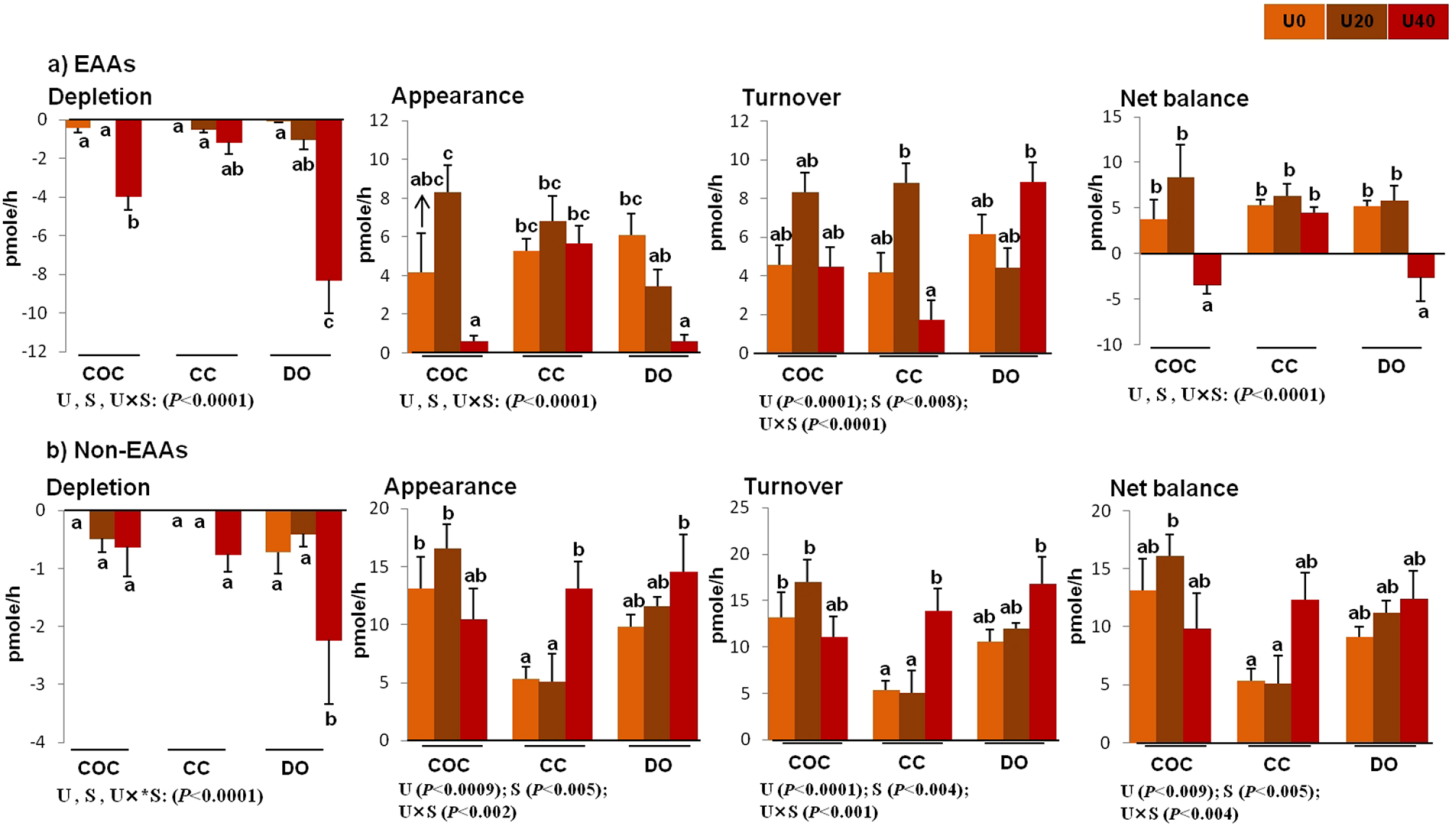
Total depletion, appearance, turnover, and net balance of essential (**a**) and non-essential (**b**) amino acids by bovine cumulus-oocyte complex, oocytectomized cumulus cells, or denuded oocytes in response to the presence of urea in maturation medium for 24 h. “Total depletion” or “total appearance” of amino acids means the sum of all negative or positive values obtained for essential amino acids. The “net balance” of amino acids was calculated by subtracting depletion from appearance, and the amino acid turnover was the sum of the net depletion and appearance. U0: control (without urea); U20: 20 mg/dl urea; U40: 40 mg/dl urea. COC: cumulus-oocyte complex; CC: cumulus cells; DO: denuded oocytes; U: the main effect of urea; S: the main effect of specimens (cell type: COCs, DOs, and CCs); U × S: interaction between urea and cell type. Data are presented as least squares means (LSM) ± standard error mean (SEM) indicated by two-way ANOVA over PROC GLM. Different letters (a, b, c, etc.) indicate significant differences between the treatments at *P* < 0.05. NS: non-significant.

There were significant interactions or main effects of urea and cell type (COCs, CCs, and DOs) on individual turnover (except threonine, which was only under the main effect of urea) (Fig. 1), total depletion, appearance, net balance, and turnover of essential amino acids (EAAs) (Fig. 2a).

Across urea concentrations, the lowest turnover of valine, histidine, methionine, tryptophan, and phenylalanine was found by COCs while DOs had the lowest turnover of leucine and isoleucine, and CCs had the lowest turnover of threonine and lysine (Fig. 1). Compared with COCs, DOs exhibited a higher total depletion (−4.38 *vs*. −9.84 pmol) or turnover (17.25 *vs*. 20.49 pmol) of EAAs (Fig. 2a).

Across cell type (COCs, CCs, or DOs), the total depletion of EAAs was higher for the 40 mg/dl urea (U40) (−13.37 pmol) compared with the 0 mg/dl urea (U0) (−0.48 pmol) and 20 mg/dl urea (U20) (−2.38 pmol). Furthermore, compared withU0, U40 negatively increased the total net balance of EAAs (14.99 *vs*. −6.23 pmol) (Fig. 2a).

### Turnover of non-essential amino acids

**Figure 3:**
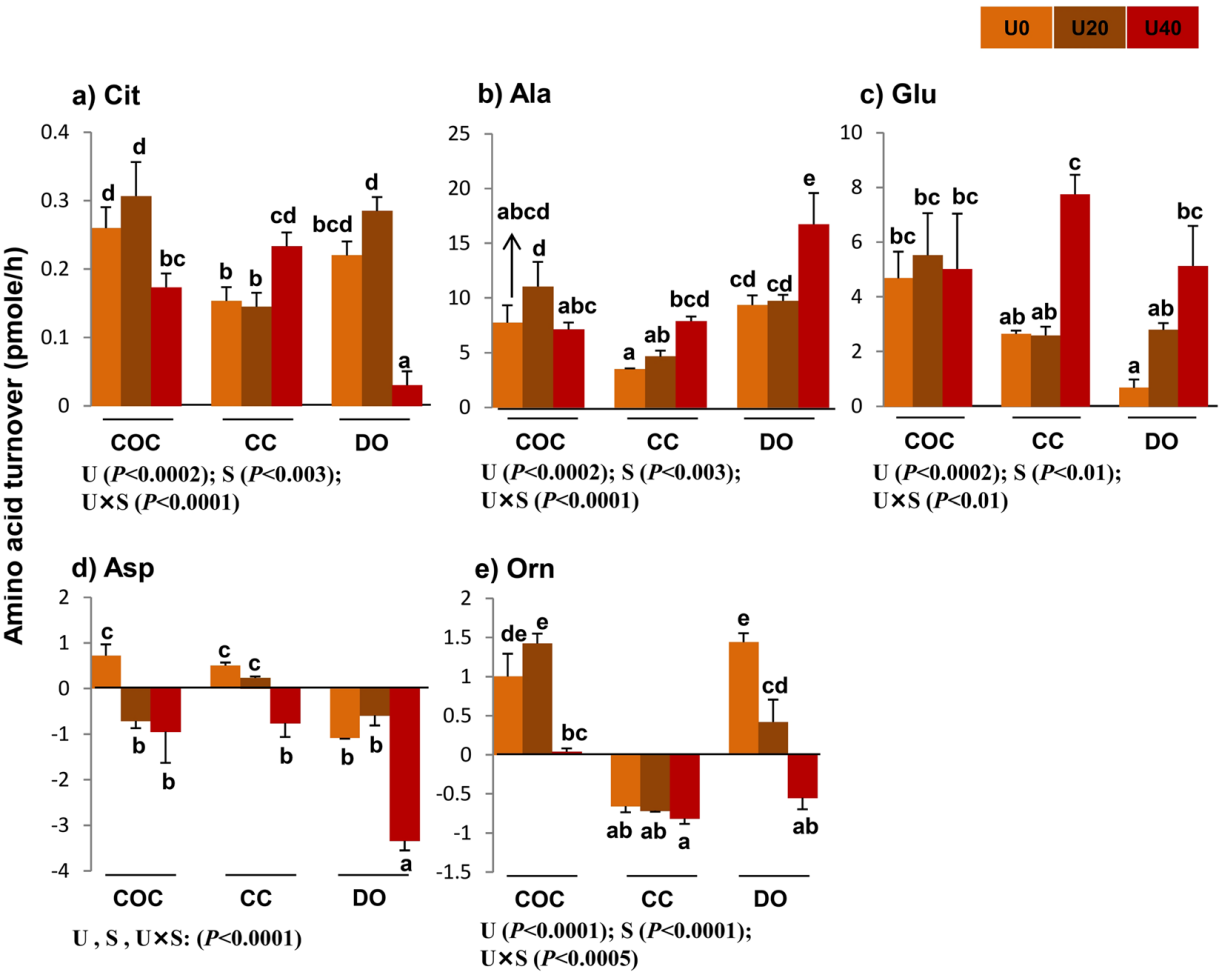
Effect of urea supplementation (0, 20, or 40 mg/dl) in maturation medium on the net depletion/appearance of non-essential amino acids by bovine cumulus-oocyte complex, oocytectomized cumulus cells, or denuded oocytes after 24-h incubation. U0: control group (without urea); U20: 20 mg/dl urea; U40: 40 mg/dl urea. COC: cumulus-oocyte complex; CC: cumulus cells; DO: denuded oocytes; U: the main effect of urea; S: the main effect of specimens (cell type: COCs, DOs, and CCs); U × S: interaction between urea and cell type. Data are presented as least squares means (LSM) ± standard error mean (SEM) indicated by two-way ANOVA over PROC GLM. Different letters (a, b, c, etc.) indicate significant differences between the treatments at *P* < 0.05. NS: non-significant.

The main effects and interaction of urea and cell type (COCs, CCs, and DOs) on individual turnover of non-EAAs (Fig. 3), total depletion, appearance, net balance, and turnover of non-EAAs were significant (Fig. 2b).

Across urea concentrations, the depletion of aspartic acid was higher in DOs (−5.02 pmol) compared with COCs (−0.93 pmol) and CCs (−0.02 pmol). DOs showed the highest appearance of alanine compared with COCs and CCs (35.82 *vs*. 25.9 and 16.06 pmol) (Fig. 3). The total depletion of non-EAAs was higher in DOs (−3.73 pmol) compared with COCs (−1.36 pmol) and CCs (−0.82 pmol). However, the appearance of non-EAAs was higher in COCs (39.82 pmol) compared with CCs (23.54 pmol) and DOs (35.16 pmol) (Fig. 2b).

Across cell type (COCs, CCs, or DOs), U40 increased the depletion of aspartic acid (−5.04 pmol) compared with U0 (0.14 pmol) and U20 (−1.07 pmol). U40 increased the appearance of alanine and glutamic acid compared with U0 (31.73 *vs*. 20.61 and 17.90 *vs*. 8.0 pmol, respectively) and U20 (25.44 and 10.91 pmol) (Fig. 3). Compared with U0 and U20, U40 increased total depletion (−0.78, −1.19, and −3.94 pmol, respectively), appearance (28.4, 31.55, and 38.57 pmol, respectively), net balance (27.62, 30.36, and 34.63 pmol, respectively), and turnover (29.18, 32.74, and 42.51 pmol, respectively) of non-EAAs (Fig. 2b).

### Turnover of semi-essential amino acids

**Figure 4:**
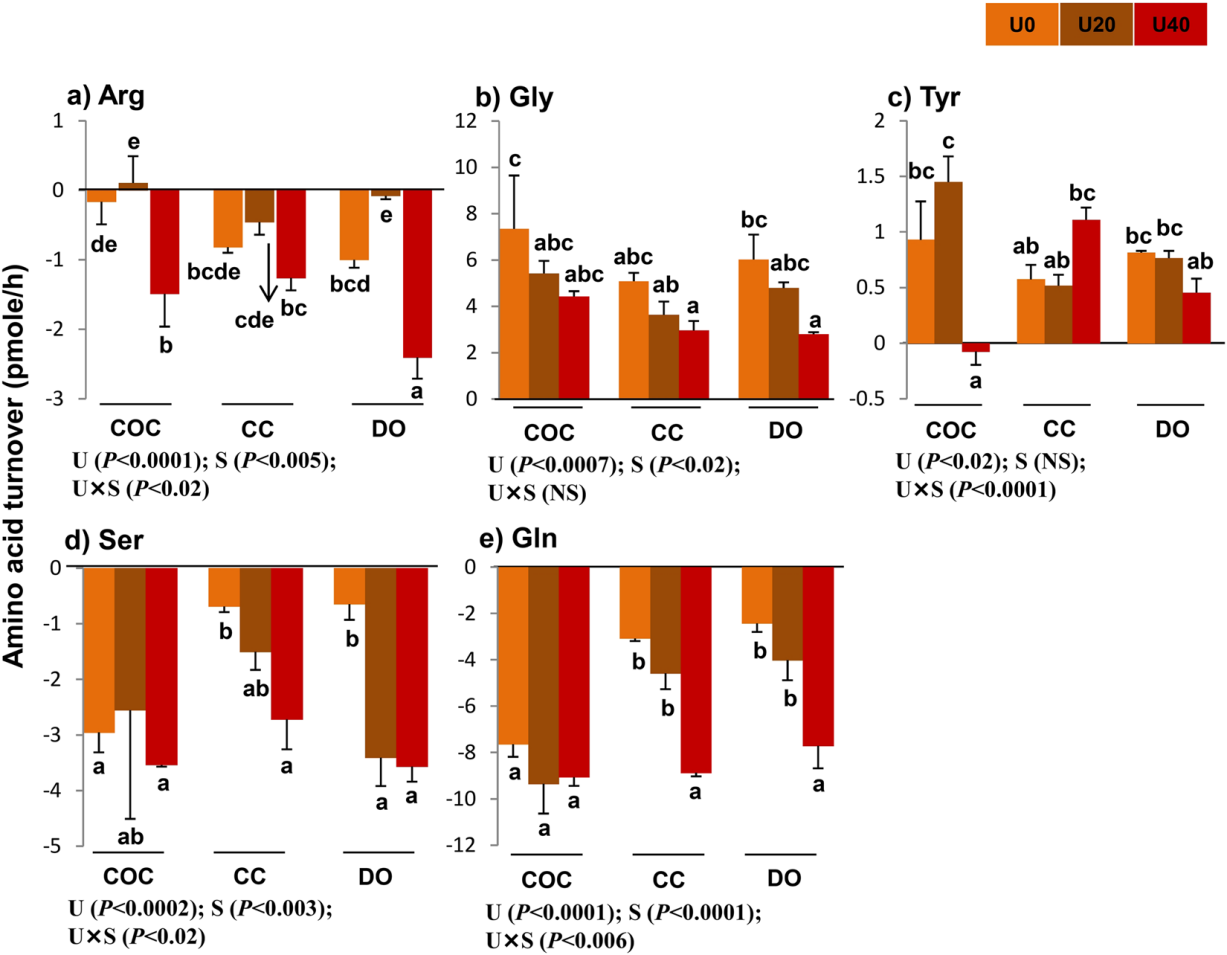
Effect of urea supplementation (0, 20, or 40 mg/dl) in maturation medium on the net depletion/appearance of semi-essential amino acids by bovine cumulus-oocyte complex, oocytectomized cumulus cells, or denuded oocytes after 24-h incubation. U0: control group (without urea); U20: 20 mg/dl urea; U40: 40 mg/dl urea. COC: cumulus-oocyte complex; CC: cumulus cells; DO: denuded oocytes; U: the main effect of urea; S: the main effect of specimens (cell type: COCs, DOs, and CCs); U × S: interaction between urea and cell type. Data are presented as least squares means (LSM) ± standard error mean (SEM) indicated by two-way ANOVA over PROC GLM. Different letters (a, b, c, etc.) indicate significant differences between the treatments at *P* < 0.05. NS: non-significant.

**Figure 5:**
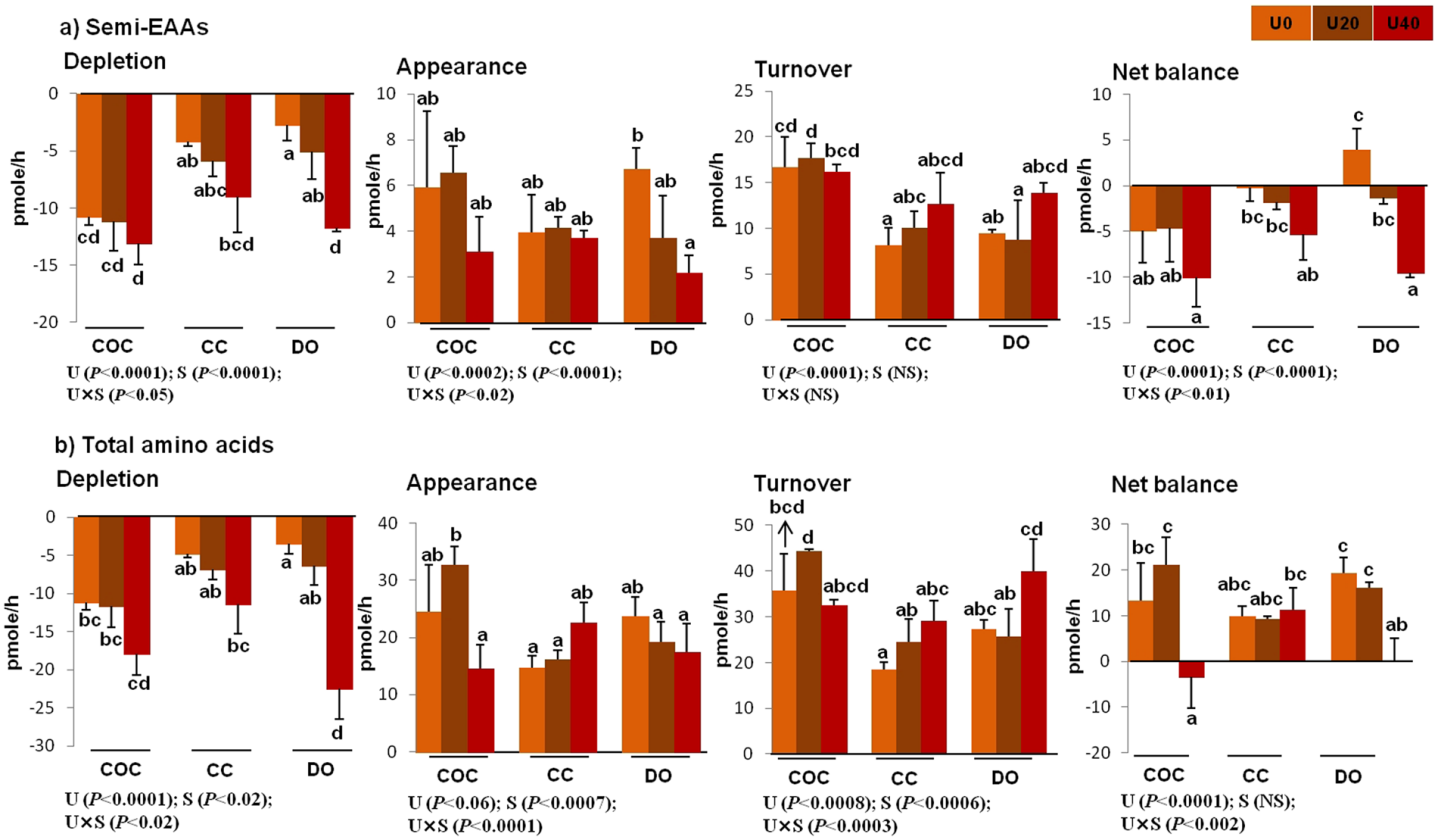
Total depletion, appearance, turnover, and net balance of semi-essential amino acids (**a**), as well as total amino acids (**b**), by bovine cumulus-oocyte complex, oocytectomized cumulus cells, or denuded oocytes in response to the presence of urea in maturation medium for 24 h. “Total depletion” or “total appearance” of amino acids means the sum of all negative or positive values obtained for non-essential amino acids. The “net balance” of amino acids was calculated by subtracting depletion from appearance, and the amino acid turnover was the sum of net depletion and appearance. U0: control (without urea); U20: 20 mg/dl urea; U40: 40 mg/dl urea. COC: cumulus-oocyte complex; CC: cumulus cells; DO: denuded oocytes; U: the main effect of urea; S: the main effect of specimens (cell type: COCs, DOs, and CCs); U × S: interaction between urea and cell type. Data are presented as least squares means (LSM) ± standard error mean (SEM) indicated by two-way ANOVA over PROC GLM. Different letters (a, b, c, etc.) indicate significant differences between the treatments at *P* < 0.05. NS: non-significant.

The interaction and main effects of urea and cell type (COCs, CCs, and DOs) on the turnover of semi-EAAs, including arginine, serine, and glutamine, were significant (Fig. 4). The turnover of glycine was under the main effects of urea and cell type, but not urea × cell type interaction. The effect of interaction between urea × cell type and the main effect of urea was significant on the turnover of tyrosine (Fig. 4). In addition, the main effects and interaction of urea and cell type on total depletion, appearance, and net balance of semi-EAAs were significant (Fig. 5a). The total turnover of semi-EAAs was only under the main effect of urea (Fig. 5a).

Across urea concentrations, the depletion of serine or glutamine was higher in COCs (−9.06 and −26.08 pmol) than that in CCs (−4.92 and −16.54 pmol) or DOs (−7.66 and −14.16 pmol). However, arginine depletion was higher in DOs (−3.49 pmol) compared with COCs (−1.55 pmol) or CCs (−2.52 pmol). The appearance of glycine was higher in COCs (17.19 pmol) compared with CCs (11.68 pmol) or DOs (13.60 pmol) (Fig. 4). The total depletion of non-EAAs was higher in COCs (−35.05 pmol) compared with DOs (−23.85 pmol) and CCs (−20.80 pmol). These results were reflected in the higher total depletion, appearance, net balance, and turnover of semi-EAAs in COCs (−35.05, 15.18, −19.87, and 50.23 pmol, respectively) compared with CCs (−20.80, 12.12, −8.68, and 32.92 pmol, respectively) and DOs (−23.85, 13.37, −10.48, and 37.22 pmol, respectively) (Fig. 5a).

Across cell type (COCs, CCs, or DOs), the depletion of serine, arginine, and glutamine was higher for U40 (−9.83, −5.16, and −25.66 pmol, respectively) compared with U0 (−4.30, −1.97, and −13.16 pmol, respectively) (Fig. 4). These results were pronounced in the higher total depletion, net balance, and turnover of semi-EAAs in U40 (−36.47, −26.90, and 46.04 pmol, respectively) compared with U0 (−19.40, −2.58, and 36.22 pmol, respectively) and U20 (−23.83, −9.55, and 38.11 pmol, respectively) (Fig. 5a). The total appearance of all semi-EAAs was higher in U0 (16.82 pmol) compared with U20 (14.28 pmol) and U40 (9.57 pmol) (Fig. 5a).

### Turnover of amino acids in COCs

Compared with U0-COCs, U20-exposed COCs released more phenylalanine (*P* < 0.0001), valine (*P* < 0.003), isoleucine (*P* < 0.03), leucine (*P* < 0.02), and methionine (*P* < 0.03) (Fig. 1a,c,f,g,d) and depleted more aspartic acid (*P* < 0.003) (Fig. 3d). In comparison with U0-COCs, U40-exposed COCs significantly depleted EAAs, including phenylalanine (*P* < 0.0004), valine (*P* < 0.03), isoleucine (*P* < 0.01), leucine (*P* < 0.02), methionine (*P* < 0.01) and lysine (*P* < 0.004) (Fig. 1a,c,f,g,d,i), non-EAAs, such as aspartic acid (*P* < 0.0009) (Fig. 3d), and semi-EAAs, such as arginine (*P* < 0.007) and tyrosine (*P* < 0.01) (Fig. 4a,c).

Compared with U20-exposed COCs groups, U40-exposed COCs significantly depleted EAAs, including phenylalanine (*P* < 0.0001), valine (*P* < 0.0001), tryptophan (*P* < 0.05), isoleucine (*P* < 0.0004), leucine (*P* < 0.0003), methionine (*P* < 0.0003) and lysine (*P* < 0.0007) (Fig. 1a,c–i), and semi-EAAs, including arginine (*P* < 0.002) and tyrosine (*P* < 0.001) (Fig. 4a,c). Compared with U0- or U20-exposed COCs, U40-exposed COCs significantly showed the highest total depletion, and lowest total appearance and net balance for EAAs (Fig. 2a) (P < 0.05).

### Urea changes amino acid turnover (depletion/appearance) by bovine CCs

Compared with U0-CCs group, U20-exposed CCs produced greater amounts of EAAs, including phenylalanine (*P* < 0.003), valine (*P* < 0.008) and methionine (*P* < 0.05) (Fig. 1a,c,d). In comparison with the U0-CCs group, U40-exposed counterparts released more EAAs, such as phenylalanine (*P* < 0.004), valine (*P* < 0.005), leucine (*P* < 0.05) and methionine (*P* < 0.03) (Fig. 1a,c,d,g), and non-EAAs, including citrulline (*P* < 0.05), alanine (*P* < 0.05), and glutamic acid (*P* < 0.0001) (Fig. 3a–c). U40-exposed CCs enhanced the production/appearance of leucine (*P* < 0.03) (Fig. 1g), citrulline (*P* < 0.05), and glutamic acid (*P* < 0.01) (Fig. 3a,c) and reduced appearance of threonine (*P* < 0.03) (Fig. 1b) compared with U20-exposed CCs,

The depletion of AAs, such as isoleucine (Fig. 1f), aspartic acid (Fig. 3d), and glutamine (Fig. 4e), was greater in U40-exposed CCs than that in U0- or U20-exposed CCs (*P* < 0.05). Compared with U0-CCs, U40-exposed CCs depleted more isoleucine and lysine (*P* < 0.07) (Fig. 1f,i) and aspartic acid (*P* < 0.006) (Fig. 3d). Compared with U20-exposed CCs, U40-exposed peers had lower total turnover of EAAs (*P* < 0.04) (Fig. 2a) and higher total appearance (*P* < 0.05) and turnover (*P* < 0.05) of non-EAAs (Fig. 2b). Moreover, in comparison with U0-CCs, U40-exposed CCs exhibited a higher total appearance and turnover of non-EAAs (*P* < 0.05) (Fig. 2b).

### Urea changes amino acid turnover (depletion/appearance) by Dos

Compared with U0-DOs, the U40-exposed counterparts significantly (*P* < 0.05) released lower amounts of EAAs, including phenylalanine (*P* < 0.002) and valine (*P* < 0.0001) (Fig. 1a,c), non-EAAs, such as citrulline (*P* < 0.001) (Fig. 3a), and semi-EAAs such as glycine (*P* < 0.03) (Fig. 4b). U40-exposed DOs produced more non- EAAs, such as alanine (*P* < 0.003) and glutamic acid (*P* < 0.0002) (Fig. 3b,c). They depleted more EAAs, such as tryptophan (*P* < 0.0001), histidine (*P* < 0.0001), isoleucine (*P* < 0.0001), methionine (*P* < 0.04) and lysine (*P* < 0.002) (Fig. 1d,e,f,h,i), non- EAAs, such as aspartic acid (*P* < 0.0002) and ornithine (*P* < 0.0003) (Fig. 3d,e), and semi-EAAs, such as arginine (*P* < 0.008), serine (*P* < 0.02), and glutamine (*P* < 0.0002) (Fig. 4a,d,e).

In comparison with U20-exposed DOs, the U40-exposed counterparts significantly depleted EAAs, such as threonine (*P* < 0.007), tryptophan (*P* < 0.0001), histidine (*P* < 0.0001), isoleucine (*P* < 0.05) and leucine (*P* < 0.008) (Fig. 1b,e–h), non-EAAs, such as aspartic acid (*P* < 0.0001) and ornithine (*P* < 0.03) (Fig. 3d,e), and semi-EAAs, including arginine (*P* < 0.0001) and glutamine (*P* < 0.004) (Fig. 4a,e) in greater quantities, while producing lower amounts of citrulline (*P* < 0.0001) (Fig. 3a) and higher alanine (*P* < 0.005) (Fig. 3b). Compared to the U0-DOs group or U20-exposed DOs, the U40-exposed DOs had a higher total depletion of EAAs (Fig. 2a), non-EAAs (Fig. 2b) and semi-EAAs (Fig. 5a), while showing the lowest appearance and net balance of EAAs (Fig. 2a) and semi-EAAs (Fig. 5a) (P < 0.05).

### Total appearance, depletion, net balance, and turnover of amino acids by COCs, CCs, or DOs cultured in the presence of different levels of urea

To calculate “total depletion” or “total appearance”, we respectively summed all negative or positive values obtained for AAs turnover^9–11^. The “net balance” of AAs was calculated by subtracting “depletion” from “appearance”. AAs turnover was calculated by the sum of “net depletion” and “net appearance”.

As shown in Fig. 5b, the total depletion, appearance, and turnover of amino acids were significantly influenced by the main effects of urea and cell type (COCs, CCs, or DOs) and their interaction. There were significant effects of urea and urea × cell type interaction on the total net balance of amino acids. Across urea concentrations, the total depletion, appearance, and turnover of amino acids were higher in COCs (−40.7, 72.39, and 113.09 pmol, respectively) compared with those in CCs (−22.07, 54.61, and 76.68 pmol, respectively) and DOs (−32.69, 61.97, and 96.73 pmol, respectively) (Fig. 5b). Across cell type (COCs, CCs, or DOs), the total depletion and turnover of amino acids were higher in U40 (−50.40 and 106.34 pmol, respectively) than those in U20 (−24.65 and 95.92 pmol, respectively) and U0 (−20.41 and 84.24 pmol, respectively) (Fig. 5b).

### Marginal effects analysis to evaluate the sensitivity of amino acids turnover to urea

**Figure 6:**
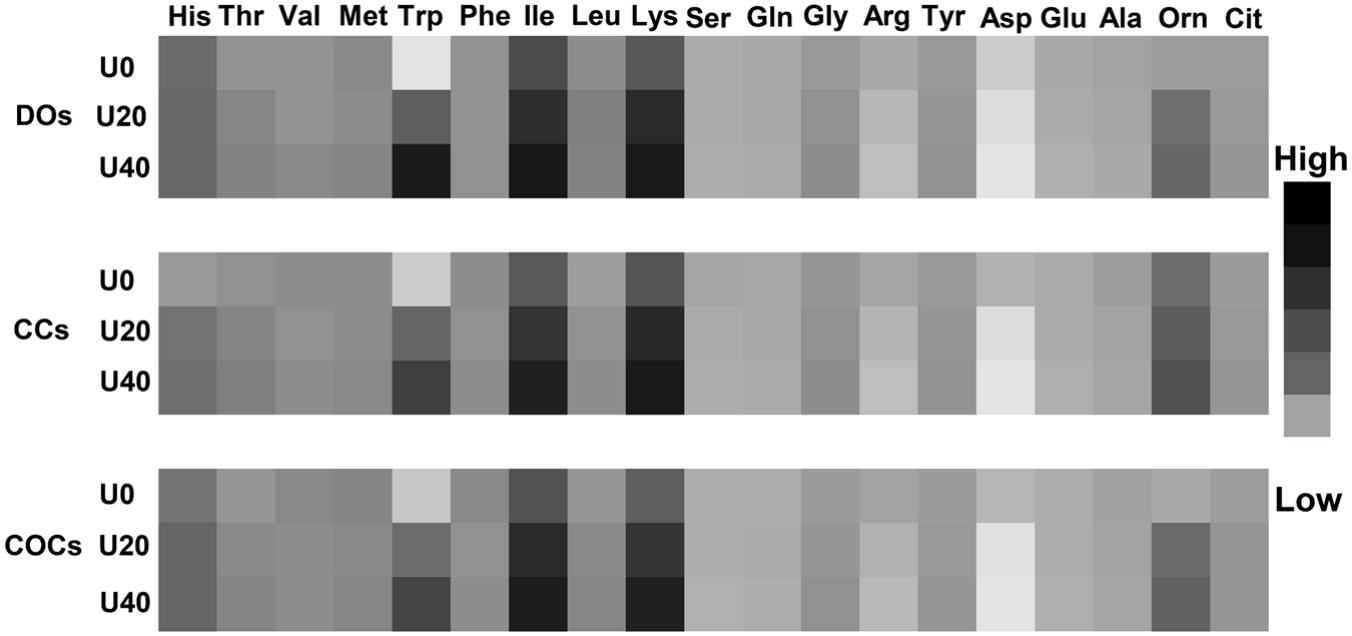
Marginal effects analysis to evaluate the sensitivity of amino acid depletion to the different levels of urea. Marginal effects analysis can detect how smaller changes in urea concentration (one-tenth intervals from 0 to 40 mg/dl) affect instantaneous changes in amino acid turnover. Marginal effects analysis determined that the turnover of isoleucine, lysine, and tryptophan were more sensitive to urea and estimated that the sensitivity of these amino acids to urea was greater in DOs (darker squares). U0: control (without urea); U20: 20 mg/dl urea; U40: 40 mg/dl urea. COCs: cumulus-oocyte complex; CCs: cumulus cells; DOs: denuded oocytes. “High” and “low” mean “greater” and “lower” turnover to urea, respectively.

A sensitivity analysis, the marginal effects, can estimate how robust an exposure (input variable, i.e., urea) may change the outcome (i.e., amino acid turnover) resulted from the experimental data in order to elucidate exposure-outcome relationships^19,20^. So, in order to predict how smaller changes in urea concentration (one-tenth intervals from 0 to 40 mg/dl) affect instantaneous change in amino acid turnover, marginal effects analysis was performed. The white and black cells in Fig. 6 are based on the values estimated by marginal effects analysis. As shown, the marginal effect analysis evaluated the higher sensitivity of isoleucine, lysine, tryptophan, and ornithine to the different levels of urea. This means that, with increasing urea level, the turnover of these AAs will be increased to a greater extent. Importantly, the increase in the sensitivity of these AAs to urea was higher in DOs (darkest squares) compared with COCs or CCs groups (Fig. 6).

### Association between amino acid turnover and developmental competence of COCs by PCA

Compared with U0-COCs, U40-exposed COCs had an increased (by 100%) rate of PN-0 (oocytes that did not reach the 2-PN stage) (*P* < 0.05). Furthermore, the subsequent cleavage, blastocyst, and hatching rates of U40- exposed COCs were reduced (*P* < 0.05) (Table 1).

**Table 1:** Developmental competence of cumulus oocyte complexes and denuded oocytes, and the viability of cumulus cells in response to supplementation of maturation medium with the different levels of urea.

Urea concentration	PN-0% 18 hours PI	Cleav. rate% (8–16 cell stage), Day 3 PI		Blast. rate% Day 7 PI	Hatch. rate% Day 9 PI	Viability% 24 hours
	COCs	COCs	DOs	COCs	COCs	CCs
0 mg/dl	12.9 ± 0.46^a^	46.2 ± 4.02^a^	16.7 ± 1.42^b^	29.1 ± 1.71^a^	28.4 ± 2.28^a^	75.6 ± 2.33^c^
20 mg/dl	17.1 ± 3.78^ab^	37.2 ± 4.42^ab^	4.43 ± 1.07^a^	22.0 ± 0.69^b^	21.1 ± 1.91^ab^	55.3 ± 2.60^b^
40 mg/dl	24.9 ± 2.78^b^	35.2 ± 2.10^b^	3.1 ± 1.88^a^	16.7 ± 2.51^c^	16.1 ± 2.48^bc^	17.7 ± 1.45^a^

Respectively, 210, 230, and 235 cumulus oocyte complexes (COCs) and 180, 210, and 200 denuded oocytes (DOs) were randomly allocated to the groups supplemented with 0, 20, or 40 mg/dl urea. Cumulus cells (CCs) had a density of 2 × 10^5^ cells/ml. The cleavage rate (8–16 cell stage, day 3 post-insemination), blastocyst rate (day 7 post-insemination), and hatching rate (day 9 post-insemination) were recorded based on the number of COCs or DOs cultured in the maturation medium. The viability of 24-h cultured CCs under different levels of urea was evaluated using Trypan blue staining. PI: post-insemination; Cleav: cleavage; Blast: blastocyst; Hatch: hatching. Different letters (a, b, c, etc.) within a column indicate significant differences at *P* < 0.05. Data were presented as mean ± SEM indicated by one-way ANOVA followed by Tukey’s multiple comparisons test.

**Figure 7:**
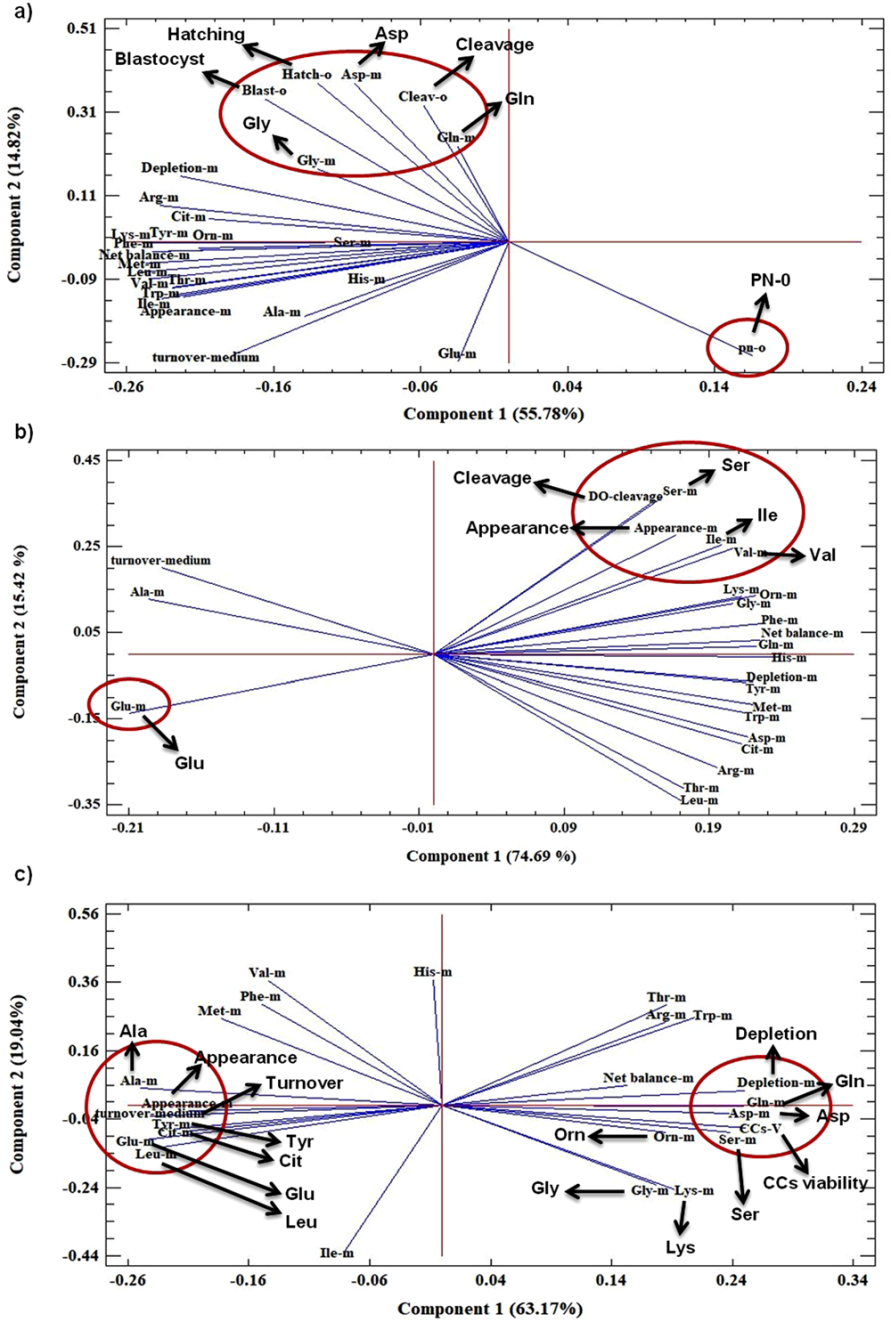
Biplot of PCA derived from the amino acid turnover and developmental competence of COCs (**a**), cleavage rate of DOs (**b**), and cell viability of CCs (**c**). Vectors with close angles (<45°) indicate a strong correlation, vectors that are perpendicular indicate no correlation, and vectors in opposite directions (approaching 180°) indicate a negative correlation.

A PCA biplot can be used to present a graphical representation of the connections between two sets of data^21^. So, we evaluated the association between amino acid turnover and developmental competence of COCs using PCA (Fig. 7a). In the biplot, the first two principal components explained 70.6% of the variance, 55.8% from PC1 and 14.8% from PC2 (Fig. 7a). PCA revealed that the turnover of glutamine and aspartic acid were positively correlated with subsequent cleavage, blastocyst and hatching rates of COCs (directional vectors at <45°), while the turnover of glutamic acid was negatively associated with them (directional vectors approaching 180°). The turnover of glycine and rates of blastocyst and hatching exhibited a strong positive association (directional vectors at <45°). The turnover of glycine, aspartic acid, arginine, citrulline and glutamine, as well as the total depletion of all amino acids, had a negative correlation with PN-0 rate (directional vectors approaching 180°) (Fig. 7a).

### Association between amino acid turnover and cleavage rate of DOs by PCA

Compared with U0-COCs, U0-DOs showed a significant reduction (from 46.2 to 16.7%) in cleavage rate (day 3 post-insemination). Interestingly, only U0-DOs reached the cleavage stage, and U20 or U40-exposed DOs arrested before reaching the cleavage stage (8–16 cell stage) (Table 1).

In the PCA biplot, the first two principal components explained 90.1% of the variance, 74.7% from PC1 and 15.4% from PC2 (Fig. 7b). PCA revealed that the turnover of serine, isoleucine and valine, as well as the total appearance of amino acids, had a positive association with the subsequent cleavage rate of DOs (directional vectors at <45°). The turnover of glutamic acid exhibited a strong negative association with the subsequent cleavage rate of DOs (directional vectors approaching 180°) (Fig. 7b).

### Association between amino acid turnover and the viability of cumulus cells by PCA

U40-exposed CCs had a significant decrease (*P* < 0.0001) in viability compared with the control group and U20-exposed CCs (Table 1). In the biplot, the first two principal components explained 82.2% of the variance, 63.2% from PC1 and 19.0% from PC2 (Fig. 7c). PCA showed that the turnover of serine, aspartic acid, ornithine, glycine, lysine and glutamine, as well as the total depletion and net balance of amino acids, had a positive association with the viability of CCs (directional vectors at <45°). The turnover of alanine, tyrosine, citrulline, glutamic acid, leucine, methionine, phenylalanine and valine, as well as the total appearance of amino acids were negatively correlated with the viability of CCs (directional vectors at 180°) (Fig. 7c).

## Discussion

In the present study, the maturation medium was supplemented with various concentrations of urea to investigate the turnover of amino acids in bovine COCs, CCs, and DOs. The urea levels were 20 and 40 mg/dl (equivalent to 9.3 and 18.7 mg/dl of the BUN, respectively). These urea concentrations were chosen to simulate BUN values usually found in healthy cows under low- or high- protein diets, respectively^7,18^. Importantly, urea concentration in the follicular fluid oscillates with a significant correlation around its normal range in serum, 16 mg/dl^3,4^. Iwata *et al*.^3^ reported that a significant association existed between urea concentration in the follicular fluid and consequent developmental competence of oocytes. Therefore, it was hypothesized that urea could differentially change amino acid turnover in bovine COCs, CCs, or DOs. In contrast to embryo^11,13,22^, little information is available on the metabolism of bovine oocyte^9,10,23^.

Data showed that the main effects of urea, cell type (COCs, CCs, or DOs), and their interaction on the turnover of most amino acids were significant. Across cell type (COCs, CCs, or DOs), 40 mg/dl urea increased the depletion of EAAs (histidine, tryptophan, lysine, isoleucine, and leucine), semi-EAAs (serine, arginine, and glutamine), and non-EAAs (ornithine and aspartic acid). These results were reflected in the increased total depletion (−20.41, −24.65, and −50.40 pmol found for 0, 20, and 40 mg/dl urea, respectively) and turnover (84.24, 95.92, and 106.34 pmol found for 0, 20, and 40 mg/dl urea, respectively) of all amino acids. Hemming *et al*.^9,10^ reported that the total turnover and depletion of amino acids was higher in incompetent oocytes than their cleaved counterparts. Therefore, it might be suggested that urea may reduce oocyte competence through increasing the need for amino acids, and total depletion and turnover of amino acids.

Across cell type (COCs, CCs, or DOs), compared with 0 and 20 mg/dl urea groups, 40 mg/dl urea increased the total appearance of alanine (20.61, 25.44, and 31.73 pmol, respectively) and depletion of glutamine (−13.16, −17.96, and −25.66 pmol, respectively). Hemmings *et al*.^9,10^ found that oocytes which failed to cleave produced more alanine and depleted more glutamine compared with those that cleaved. However, ammonium has been demonstrated to have no effect on turnover of these amino acids in bovine blastocysts^22^. It has also been shown that metabolism of glutamine to CO2 can be considered as a marker for tricarboxylic acid (TCA) cycle activity and indicates glucose oxidation within bovine COCs^23^. So, high urea level may alter the metabolism of glutamine and probably TCA cycle activity.

To obtain a better understanding of how robust urea could affect amino acid turnover, marginal effects analysis was performed^21^. Marginal effects analysis detected how smaller changes in urea concentration (one-tenth intervals from 0 to 40 mg/dl) affected instantaneous change in amino acid turnover. Marginal effects analysis evaluated the high sensitivity of isoleucine, lysine, and tryptophan turnover to urea and estimated that the sensitivity of these amino acids to urea was greater in DOs (darker squares in Fig. 6). As mentioned, the marginal effects analysis estimated that, with increasing urea concentration, the turnover of lysine was increased. In addition, the present data showed that, with increasing urea concentration, COCs and DOs significantly depleted more lysine and methionine, the most limiting AAs for milk production in lactating dairy cows^24^. Importantly, the concentrations of milk-limiting AAs in the follicular fluid of high-yielding cows is low^25^, resulting in a reduced availability of these AAs for oocytes. Coupling such situation with urea-induced consumption of these AAs may worsen the availability of milk limiting AAs for oocytes. At a minimum, the results of marginal effects can be utilized to design future experiments more efficiently. However, additional investigations to confirm these findings are requisite.

Across urea concentration, DOs released more alanine in the media compared with COCs and CCs (35.82, 25.90, and 16.06 pmol, respectively). In addition, COCs depleted more glutamine compared with CCs and DOs (−26.08, −16.54, and −14.16 pmol, respectively). In fact, mammalian oocytes require adenosine triphosphate (ATP) to carry out their meiotic resumption^26^. Within the COCs, the cumulus cells provide the required ATP for the oocyte via oxidative phosphorylation and fatty acid β-oxidation^27^. So, the higher depletion of glutamine by COCs than DOs may ensure sufficient availability of substrate to support TCA cycle activity in COCs^23^ Moreover, since oocytes do not express alanine transporter, they need CCs to support the uptake of alanine^17^. This may explain the greater alanine concentration in DOs medium than COCs.

Finally, the subsequent developmental competence of COCs and DOs matured under different concentrations of urea were recorded. Compared with COCs, DOs showed a very significant reduction in cleavage rate. Similarly, Zhang *et al*.^28^ reported that denudation of bovine oocytes before IVM substantially reduced the cleavage rate compared with denudation after IVM (5% *vs*. 47%). It has been reported that CCs keep toxic products of metabolism away and play roles in glutathione synthesis and antioxidant machinery^29,30^. This offers protection against reactive oxygen species and chemical toxins, i.e., urea, for oocyte^29,30^. These findings indicate an important physical interaction between cumulus cells and oocytes in the context of next developmental competence^30^. Furthermore, data revealed that urea dose-dependently increased the rate of COCs that remained un-fertilized (PN-0), but reduced the subsequent developmental competence of COCs. Similarly, Iwata *et al*.^3^ reported that a negative correlation exists between urea concentration in the follicular fluid and developmental competence of oocyte in bovines.

PCA analysis was carried out to identify the potential relationship between amino acids turnover (by COCs and DOs) and their subsequent developmental competence, as well as the viability of CCs. In the PCA biplot, correlations were represented by directional vectors and determined by the angle between vectors^21^. Vectors with angles <45° had a strong correlation, vectors with a perpendicular angle (approaching 90°) had no correlation, and vectors in opposite directions (approaching 180°) had a negative correlation.

The PCA biplot (Fig. 7a) revealed that the cleavage rate was positively correlated with the turnover of glutamine and aspartic acid in COCs. This means that greater glutamine and aspartic acid turnover were significantly associated with a higher cleavage rate. Similar to our results, Zuelke *et al*.^23^ reported that LH increased the metabolism of glutamine by oocyte and improved oocyte maturation. Indeed, the metabolism of glutamine to CO2 has been known as a marker for activity of the TCA cycle^23^. So, higher depletion of glutamine may ensure higher activity of the TCA cycle in COCs, and consequently its higher cleavage rate. However, Hemming *et al*.^9,10^ found that DOs that experienced fertilization and cleavage had a reduced demand for glutamine. In the present study, we found that, across urea concentrations, COCs depleted more glutamine compared with DOs (−26.08 *vs*. −14.16 pmol), implying a higher TCA cycle activity within COCs^23^. Similarly, Zuelke *et al*.^23^ reported that the activity of theTCA cycle inside DOs (denuded before IVM) was approximately half of that in COCs (denuded after IVM). This might explain the higher developmental competence of COCs versus DOs due to better energy availability in COCs.

PCA biplot detected a strong positive relationship between serine turnover and cleavage rate of DOs (Fig. 7b). As mentioned above, within COCs, CCs provide the required ATP for the oocyte *via* oxidative phosphorylation and fatty acid β-oxidation^27^. Hence, DOs may be encountered with insufficient TCA cycle activity (due to lower glutamine consumption) and ATP availability (which should be normally provided by CCs). Serine has been shown to be converted to pyruvate and support energy availability^31^. Consequently, the positive correlation of serine depletion and cleavage rate of DOs can be explained by the role of serine in providing energy as an exogenous source^22^.

PCA revealed that a strong positive or negative correlation existed between the turnover of glycine and developmental competence of COCs or rate of PN-0, respectively. Glycine has been demonstrated to be utilized by oocyte to balance external osmolarity and regulate cell volume^32^. In addition, as a part of the glutathione system, it can be involved in protection against reactive oxygen species (ROS) and apoptosis^33^. In the present study, with increasing concentrations of urea, the appearance of glycine was decreased in parallel (0 mg/dl urea: 18.46 pmol; 20 mg/dl urea: 13.84 pmol; 40 mg/dl urea: 10.84 pmol). Moreover, across urea concentration, the appearance of glycine in the medium of DOs was lower than that of COCs (13.60 *vs*. 17.19 pmol). These data suggested that, within DOs or under high urea concentration, the need for glycine was higher, and thus lower amounts of glycine were released into the medium. This also implied a higher accumulation of glycine within DOs. We recently demonstrated that urea induced shrinkage in bovine DOs^34^. So, DOs may require a greater amount of glycine for protection against osmotic stress induced by urea. However, further investigations are necessary to confirm these findings.

The present data revealed that urea reduced the viability of CCs. Similarly, we recently reported that urea decreased the viability of oviduct epithelial cells^35^. It has been shown that oocyte regulates the viability of CCs by activating the intermedin signaling pathway and expressing growth factors^16^. The present finding implied the potential role of oocyte signaling in the normal development of CCs^16^. The PCA biplot showed that serine turnover (depletion) and alanine appearance had a strong positive or negative correlation with the viability of CCs, respectively (Fig. 7c). Serine has been demonstrated to be involved in the metabolism of nucleic acids *via* the tetrahydrofolate (THF) cycle, while its impairment inhibits cell proliferation^36^. This suggests the potential role of serine in CCs viability.

As previously mentioned, CCs viability exhibited a negative association with alanine turnover (appearance). Alanine has been identified as an apoptosis marker, in which its concentration is increased in apoptotic cells^37^. This implies a relationship between lower CCs viability and higher appearance of alanine.

In conclusion, the turnover of AAs in bovine COCs, CCs, and DOs was significantly changed by urea. High urea concentration induced total depletion and turnover of amino acids and reduced consequent developmental competence of oocytes. Importantly, PCA analysis revealed a strong positive correlation between developmental competence of COCs and the turnover of glycine, aspartic acid, and glutamine. Furthermore, the rate of COCs that remained un-cleaved was negatively correlated with the turnover of glycine, aspartic acid, arginine, and glutamine. The results of the present study suggest that urea may increase the global demand of COCs for amino acids.

## Methodology

### Animal studies and data statement

Animal experiments were carried out according to the Guiding Principles for the Care and Use of Research Animals Promulgated by the Isfahan University of Technology, Iran. The protocol and methods were approved by the Committee on the Ethics of Animal Experiments of the Isfahan University of Technology. The datasets are available from the corresponding author upon request.

### COCs collection and *in vitro* maturation

COCs collection and oocyte maturation were conducted according to previous studies^38^. Briefly, using a thermo box containing 0.9% NaCl, 0.1% penicillin and streptomycin (Sigma-Aldrich, St. Louis, MO, U.S.A.), bovine ovaries were transported from a local abattoir to the laboratory within 2 h at approximately 30 °C. The ovaries were washed in 30 °C sterile 0.9% NaCl, containing 0.1% penicillin and streptomycin, and stored in sterile saline until aspiration using an 18 G needle. To reduce heterogeneity in the number of cumulus layers and the maturation stage of oocytes, only medium-size (7 to 8 mm) follicles were strictly aspirated. Importantly, stages of maturation^39^ and patterns of amino acid transporters during various stages of oocyte growth^8^ influence the response of oocyte to treatments. Hence, oocyte should be collected at a similar stage, for example, metaphase II (MII)^9,10^, or their nuclear and cytoplasmic maturation should be synchronized (using components that accumulate and induce the cAMP pathway within the oocyte)^40^. It has been shown that oocytes at the MII stage have completed the final maturation process^41,42^. In addition, the activity of some amino acid transporters can be reduced from the GV to MII stages^8^. So, as the bovine oocyte grows, its synthetic activity gradually decreases, resulting in a quiescent stage^41,43^. Moreover, cAMP has been reported to be involved in regulating the activity of amino acid transporters^44,45^. Consequently, we decided not to use MII oocytes or *in vitro* synchronization to avoid any potential changes in the activity of amino acid transporters. In order to have oocytes with as similar a maturation stage as possible, oocytes were strictly collected from 7–8 mm follicles. It has been reported that oocytes derived from follicles that are similar in size exhibit a close maturation stage^46^.

Only COCs with at least five cell layers were only selected under a stereomicroscope (Olympus, Tokyo, Japan) and transferred in HEPES-TCM-199 (tissue culture medium) (Invitrogen, Carlsbad, CA, U.S.A.) supplemented with 50 mg/ml kanamycin (Sigma-Aldrich, St. Louis, MO, U.S.A.) and 50 mg/ml heparin (Sigma-Aldrich, St. Louis, MO, U.S.A.). The intact COCs were then washed twice in HEPES-TCM199 and randomly placed in groups of 10 into a 50-µL droplet of maturation medium, bicarbonate-buffered TCM199 supplemented with 10% (w/v) heat inactivated fetal bovine serum (FBS, BioWhittaker, Walkersville, MD, U.S.A.), and 20 IU/ml follicle-stimulating hormone (FSH, Sigma-Aldrich, St. Louis, MO, U.S.A.) in 35 mm cell culture dishes (Falcon brand, BD Biosciences, San Jose, CA, U.S.A.) for 24 h at 38.5 °C under 5.5% CO_2_, 20% O_2_, balanced N_2,_ and maximum humidity under mineral oil.

A total of 675 COCs were randomly allocated to the control group (maturation medium without urea, n = 210); U20 (maturation medium supplemented with 20 mg/dl urea, n = 230; Sigma-Aldrich, St. Louis, MO, U.S.A); and U40 (maturation medium supplemented with 40 mg/dl urea, n = 235) for 24 h. Then, the 24-h spent medium was collected and stored at −70 °C.

### Oocyte denudation before IVM

Oocytes were completely denuded by vortex agitation for 1 min at 37 °C, followed by manual pipetting in HEPES-TCM-199 and centrifuged at 200 × g to separate from CCs^47^. approximately 590 DOs were assigned to the control group (maturation medium without urea, n = 180); U20 (maturation medium supplemented with 20 mg/dl urea, n = 210); and U40 (maturation medium supplemented with 40 mg/dl urea, n = 200). The DOs were cultured in maturation medium in groups of 10 under the same conditions for 24 h as for COCs culture. Then, the 24-h spent medium was collected and stored at −70 °C.

### Oocytectomy and culture of cumulus cells

The oocytectomized CCs were pooled and washed twice by centrifugation (200 × g, 5 min each) in culture medium^47,48^. The resultant pellets were then suspended in culture medium. Cumulus cells were randomly allocated to the control group (maturation medium without urea); U20 (maturation medium supplemented with 20 mg/dl urea); and U40 (maturation medium supplemented with 40 mg/dl urea) at 2 × 10^5^ cells/ml density for 24 h under the same conditions as for COCs and DOs culture. The number of cultured cumulus cells was based on the average number of CCs attached to each COC. Approximately (2.3 ± 0.4) × 10^4^ cells were attached to each COC. So, 10 oocytes per drop consisted of approximately 2 × 10^5^ cumulus cells. Therefore, we cultured cumulus cells at a density of 2 × 10^5^ cells/drop. After 24-h incubation, the viability of cultured CCs was evaluated using Trypan blue staining. Then, the 24-h spent medium was collected and stored at −70 °C.

The urea concentrations used in this study (20 mg/dl urea equivalent to 9.3 mg/dl BUN, and 40 mg/dl urea equivalent to 18.7 mg/dl BUN) were based on blood and follicular urea concentrations found in healthy dairy cows fed a low- or high-protein diet, respectively^7,18^. Importantly, the urea concentration of follicular fluid has been comparable with that of plasma^4^.

### Amino acid determination using HPLC

AAs levels were determined in the 24-h spent media (Figs 1, 3 and 4), follicular fluid collected from 7–8 mm size follicles of 10 cows, and fresh maturation media (n = 4) (see Supplementary Table S1) using the HPLC method. Previous studies evaluated the amino acid turnover in MII oocytes in bovine and human^9,10^. They measured the final 6 h of *in vitro* maturation medium with a reduced amino acid concentration (one-sixth MEM and five-sixth Earle’s Balanced Salt Solution). It has been shown that, in cultured mammalian cells, sudden reduction in nutrients, i.e., amino acids, rapidly induces autophagy within minutes^49^. It has also been shown that oocyte at the MII stage has completed the final maturation processes and its synthetic activity may gradually decrease, resulting in a quiescent stage^41–43^. However, it has been reported that the developmental competence of embryos can take advantage of the reduction in amino acid content of the culture medium. For example, Liu and Foote^50^ demonstrated that a reduction in essential amino acids concentration (by 50%) resulted in a higher developmental competence of bovine embryos. In the present study, the amino acid concentrations were not reduced in the media during *in vitro* maturation (24 h) (see Supplementary Table S1) and were based on the concentrations present in the tissue culture medium (TCM)-199 and previous works^51^. In the present study, the total amino acids concentration present in the maturation medium was higher than that detected in the follicular fluid (4.1 *vs*. 3.7 μmol/ml) (see Supplementary Table S1), while it was still comparable with other reports^51^. Nakazawa *et al*.^52^ showed that supplementation of culture medium with amino acids at concentrations similar to those found in the follicular fluid improved mouse blastocyst rate. So, we utilized 24-h spent medium for amino acid analysis to cover all of the steps of oocyte/COCs metabolism. In addition, to avoid potential induction of undesired responses, such as autophagy^49^, amino acid concentrations were not reduced in the media. To avoid any interaction with urea in the medium, the maturation media was free from bovine serum albumin (BSA). In the present study, we cultured COCs, DOs, and CCs in six replicates, such that each replicate was composed of five drops with COCs, five drops with DOs, and five drops with CCs. Prior to performing HPLC analysis, collected media were combined as replication 1 with 2; replication 3 with 4; and replication 5 with 6. Therefore, three aliquots (approximately 200 µl) were produced per replicate for HPLC analysis. The average statistical power of this experiment was sufficient^53^ at 86.1 ± 10.8%, ranging from 65.6 to 93.1%). Furthermore, there was a 95% probability that the obtained confidence interval covered the mean in all observations (see Supplementary Table S2).

The mean of each amino acid concentration in the blank droplets (maturation media with no COCs, CCs, DOs, or urea supplementation) that had been cultured in identical conditions with the test samples was used as the reference (blank) to calculate the depletion or appearance of each amino acid. After 24-h incubation, all of the blank and test media were immediately stored at −70 °C and then analyzed using the same protocol.

After thawing the spent maturation medium at room temperature, AAs concentration was measured using HPLC (HyperClone ODS C 18, 250 mm × 4.6 mm, 5 μm, Agilent 1100, Agilent Technologies, Waldbronn, Germany) at a flow rate of 1.2 ml/min with fluorescence detection after pre-column derivatization with o-phthaldialdehyde (OPA)^54^. The fluorescent signal was 450 nm when excited at 348 nm. For separation, mobile buffer A (80% 83 mM Na-acetate, 19.5% methanol, 0.5% tetrahydrofuran) and mobile buffer B (80% methanol/20% 83 mM Na-acetate) were used. Chromatography was carried out at 25 °C at a flow rate of 1.2 ml/min. Total analysis time was 50 min. To determine amino acid concentrations, peak areas of samples and standard amino acid mixture were compared. Using peak signals from the internal standard, all peak signals were normalized. The OPA method employed in the present study has been shown to have a low response to cystine and none to proline^55^. Furthermore, because of conversion of asparagine to aspartic acid during sample preparation, the assay of asparagine was impossible^55^.

Amino acids were classified as EAAs (Leu, Lys, Phe, Ile, Val, Met, Trp, Thr, and His), semi-EAAs (Ser, Arg, Tyr, Gly, and Gln), and non-AAs (Asp, Ala, Cit, Orn, and Glu)^51,56^. Ser, Gly and Gln are commonly considered as non-EAAs^56^, while these amino acids have been shown to be semi-essential for animals to accomplish their full hereditary potential for growth, development, reproduction, lactation, and resistance to metabolic and infectious diseases^57^.

### Accuracy of amino acid determination

In order to evaluate the accuracy of the amino acid analysis, we determined the mean recovery of one biological medium that contained a certain amounts of amino acids which was comparable with the amount in the maturation media. Accuracy was defined as the agreement between the measured value and actual value of amino acids in the reference media. The average recovery for amino acids for all detected amino acids was 102.6%. The accuracy ranged from 93.1% to 106.9%, except for aspartic acid (112.4%). So, the limit for all amino acids was assigned between 90% and 115% (see Supplementary Table S3). Moreover, in order to test the precision of the method, we measured repeatability through injecting the same sample of fresh maturation media (four times) in a row and calculating the relative standard deviation (*RSD*) for each amino acid. The obtained *RSD* values were in the acceptable range^58^ of 1.12 to 3.36% (see Supplementary Table S4).

### Marginal effect analysis

To determine how changes in amino acid turnover were related to changes in the input variable (urea), marginal effects were computed. In the present dose-dependent experiment, the concentrations of the variable (urea) had been increased from 0 to 20, and then to 40 mg/dl. So, the marginal effects were computed to reveal the instantaneous rate of change in amino acid turnover based on the smaller intervals (one-tenth fold intervals from the lowest to the highest urea concentration, 0 to 40 mg/dl)^19,20^. This method provides a good estimate of the amount of change in amino acid turnover that will be produced by a one-unit change (i.e., one-tenth fold intervals) in the variable, i.e., urea^59^. In the present study, the turnover of AAs depended on urea concentrations (0, 20, and 40 mg/dl) as a continuous variable and on cell type, i.e., CCs, DOs, and intact COCs as discrete variables. To estimate the effects of these variables on AAs turnover, the marginal effects of these variables were used by the following relation for discrete variables of CCs and DOs: $E({\rm{\Delta }}A)=E(Ai|{\boldsymbol{X}}i,\,{\boldsymbol{\beta }}-Ai|{\boldsymbol{X}}i=1,\,{\boldsymbol{\beta }})$, in which $Ai|{\boldsymbol{X}}i,\,{\boldsymbol{\beta }}$ was the predicted AAs turnover using the response surface model (RSM) that was calibrated using the excremental of the dataset. $Ai|{\boldsymbol{X}}i=1,\,{\boldsymbol{\beta }}$ was the predicted data *i*-th for the dataset. All discrete variables were considered as 1. *E*(Δ*A*) was the mean difference between the predicted AAs turnover and the predicted RSM using the input data set with CCs and DOs equal to 1. *Xi* = 1 was the different values for the continuous variable of urea and estimated as $E({\rm{\Delta }}A)=E(\frac{Ai|{\boldsymbol{X}}i,\,{\boldsymbol{\beta }}-Ai|{\boldsymbol{X}}i+{\rm{\Delta }}{\boldsymbol{X}}i,\,{\boldsymbol{\beta }}}{{\rm{\Delta }}{\boldsymbol{X}}i})$, in which Δ*Xi* was a small value for each urea level. Three levels of urea as ***X****i* = 0, 20, or 40 were investigated in the current study using RSM by the following function^60^: $A|{\boldsymbol{X}}={\beta }_{0}+\sum _{i=1}^{n}{\beta }_{i}{x}_{i}+\sum _{i=1}^{n}\sum _{j\ge i}^{n}{\beta }_{ij}{x}_{i}{x}_{j}$, in which *β*_0_, *β*_*i*_, and *β*_*ij*_ were the unknown coefficients which could be calibrated using the dataset of the AAs turnover based on the input dataset of urea, COCs, CCs, or DOs. The unknown coefficients were estimated using the least square method.

### *In vitro* fertilization and embryo culture

After *in vitro* maturation, urea-exposed COCs/DOs and control COCs/DOs (n = 10 per droplet) were fertilized with sperm cells at a final concentration of 1 × 10^6^/ml in *in vitro* fertilization medium (50-μl droplets) supplemented with BSA in 35 mm cell culture dishes (Falcon brand, BD Biosciences, San Jose, CA, U.S.A.) at 38.5 °C under 5% CO_2_ in air. Sperm capacitation was performed using a modified Tyrod’s albumin, lactate, and pyruvate medium (Sp-TALP) in a 96-well untreated polystyrene microplate (Corning Incorporated, New York, U.S.A.)^61^. In brief, after 20 min swim-down, sperm was suspended in Sp-TALP supplemented with 10 mg/ml heparin (50 × 10^6^ sperm/ml) and incubated for 18 h. Zygotes were recovered 24 h post-insemination, and CCs were manually stripped from the oocytes by repeat pipetting using narrow-bore glass pipettes in 80 IU/ml bovine hyaluronidase in HEPES-buffered MEM (BioWhittaker, Walkersville, MD, U.S.A.). Resultant embryos were transferred into 35 mm cell culture dishes (n = 6 per droplet) containing synthetic oviduct fluid medium (20-μl droplets) supplemented with 30 µl/ml of EAAs solution (50×; 11130-051; Gibco), 10 µl/ml of non-EAAs solution (100×; 11140; Gibco), and 4 mg/ml BSA in an incubator at 38.5 °C, 5% O_2_, 6% CO_2_, and 90% N_2_. The rate of COCs that did not reach the two-PN stage (PN-0) was recorded at 18 h post-insemination using a stereomicroscope (Olympus, Tokyo, Japan). Cleavage rate (8–16 cell stage, day 3 post-insemination), blastocyst rate (day 7 post-insemination), and hatching rate (day 9 post-insemination) were recorded based on the initial number of COCs or DOs cultured in the maturation medium.

### Statistical analysis

The normality of all data was examined prior to analysis using the Anderson-Darling test (EasyFit software, version 5.6, MathWave Technologies, Spokane, WA, U.S.A.). The data of amino acid turnover and the rate of COCs that failed to reach the two-PN stage (PN-0), cleavage rate, blastocyst rate, and hatching rate exhibited a normal distribution. Statistical analysis was performed using StatView 5.0 (SAS Institute Inc., Cary, NC, U.S.A.) or MATLAB software (Version 7.10.0; MathWorks Inc., Natick, MA, U.S.A.). The data of fertilization and developmental competence were analyzed using the one-way ANOVA followed by multiple comparisons test, Tukey’s *post hoc* analysis, and these data were presented as mean ± standard error mean (SEM). The main effects of urea and cell type (COCs, DOs, and CCs), and the interaction between urea and cell type on amino acid turnover were evaluated using two-way ANOVA over PROC GLM, and these data were presented as least squares means (LSM) ± SEM. The results were considered as statistically significant at *P* < 0.05. Principal component analysis (PCA) was performed to identify the association of amino acid turnover with cumulus cell viability or developmental competence of COCs and DOs (regardless of urea treatment). Multiple dimensions of the data were reduced to two dimensions, and the biplot was prepared using Statgraphics Centurion 16.1.11 (Statpoint Technologies, Inc., Warrenton, U.S.A.).

## Electronic supplementary material

Supplementary Information
