# Supplementary material for: Urea influences amino acid turnover in bovine cumulus-oocyte complexes, cumulus cells and denuded oocytes, and affects in vitro fertilization outcome
Source: Sci Rep. 2018 Aug 15;8:12191. doi: 10.1038/s41598-018-30774-2 (PMC6093885; doi:10.1038/s41598-018-30774-2)
Supplement: Supplementary file 1 — Supplementary Information [file 41598_2018_30774_MOESM1_ESM.pdf]

Supplementary Information, SREP-18-14669.

Urea influences amino acid turnover in bovine cumulus-oocyte complexes, cumulus cells and denuded oocytes, and affects *in vitro* fertilization outcome

Rasoul Kowsar[1,2*],Vahid Norozian Iranshahi[1], Nima Sadeghi[3], Ahmad Riasi[1], and Akio Miyamoto[2]

[1] Department of Animal Sciences, College of Agriculture, Isfahan University of Technology, Isfahan, 84156–83111, Iran

[2] Graduate School of Animal and Food Hygiene, Obihiro University of Agriculture and Veterinary Medicine, Obihiro, Hokkaido 080-8555, Japan

[3] FKA, Animal Husbandry and Agriculture Co., Isfahan, Iran

**Amino acid concentration** (μmol/ml)	**Maturation media, n=4**	**Follicular fluid, n=10**
**Leu**	0.36 ± 0.007[b]	0.17 ± 0.017[a]
**Lys**	0.34 ± 0.007[b]	0.23 ± 0.077[a]
**Phe**	0.12 ± 0.002[a]	0.15 ± 0.011[a]
**Ile**	0.23 ± 0.004[b]	0.15 ± 0.014[a]
**Val**	0.16 ± 0.003[a]	0.27 ± 0.022[b]
**Met**	0.08 ± 0.001[b]	0.02 ± 0.002[a]
**Trp**	0.04 ± 0.001[a]	0.06 ± 0.002[b]
**Thr**	0.17 ± 0.006[b]	0.13 ± 0.004[a]
**His**	0.09 ± 0.002[b]	0.08 ± 0.005[a]
**Ser**	0.18 ± 0.003[b]	0.14 ± 0.005[a]
**Arg**	0.27 ± 0.004[a]	0.20 ± 0.040[a]
**Tyr**	0.18 ± 0.003[b]	0.12 ± 0.012[a]
**Gly**	0.62 ± 0.009[a]	0.63 ± 0.064[a]
**Gln**	0.40 ± 0.006[a]	0.58 ± 0.010[b]
**Asp**	0.15 ± 0.003[b]	0.02 ± 0.002[a]
**Cit**	0.02 ± 0.001[a]	0.04 ± 0.014[b]
**Ala**	0.32 ± 0.006[a]	0.50 ± 0.017[b]
**Orn**	0.02 ± 0.001[a]	0.03 ± 0.020[a]
**Glu**	0.40 ± 0.006[b]	0.22 ± 0.058[a]
**Total**	4.17 ± 0.062[b]	3.70 ± 0.077[a]

Supplementary Table S1. Amino acid concentration in the follicular fluid and fresh maturation medium measured by HPLC. Different letters (a, b, c, etc) indicate significant differences between the treatments at $P < 0.05$. Data were presented as mean ± SEM.

Amino acid	DOs			CCs			COCs		
	U0	U20	U40	U0	U20	U40	U0	U20	U40
Asp	-1.09<br>(-1.12 , -1.05)	-0.59<br>(-1.14, -0.04)	-3.34<br>(-3.89, -0.79)	0.51<br>(0.34, 0.67)	0.23<br>(0.11, 0.36)	-0.76<br>(-2.08, 0.56)	0.72<br>(-0.33, 1.78)	-0.71<br>(-1.11, -0.31)	-0.95<br>(-2.65, 0.76)
Glu	0.675<br>(-0.08, 1.43)	2.80<br>(2.19, 3.40)	5.13<br>(4.52, 5.74)	2.64<br>(2.12, 3.17)	2.59<br>(1.79, 3.39)	7.75<br>(5.99, 9.51)	4.68<br>(0.51, 8.85)	5.52<br>(-1.12, 12.15)	5.02<br>(0.0, 10.04)
Ser	-0.65<br>(-1.35, 0.05)	-3.42<br>(-4.66, -2.18)	-3.57<br>(-4.81, -0.33)	-0.69<br>(-0.94, -0.44)	-1.51<br>(-2.31, -0.70)	-2.72<br>(-4.06, -1.38)	-2.96<br>(-4.50, -1.42)	-2.56<br>(-7.40, 2.29)	-3.54<br>(-3.61, -3.47)
Gln	-2.43<br>(-3.6, -1.3)	-4.02<br>(-6.2, -1.9)	-7.71<br>(-9.9, -5.6)	-3.08<br>(-3.6, -2.6)	-4.58<br>(-7.5, -1.6)	-8.88<br>(-9.3, -8.5)	-7.65<br>(-10.0, -5.3)	-9.36<br>(-14.9, -3.8)	-9.07<br>(-10.7, -7.5)
His	0.38<br>(-0.32, 1.08)	-0.48<br>(-0.53, -0.43)	-3.71<br>(-3.76, -3.66)	2.35<br>(1.35, 3.35)	3.07<br>(0.94, 5.2)	2.63<br>(2.43, 2.83)	0.07<br>(-0.9, 1.04)	0.57<br>(-0.33, 1.46)	0.13<br>(-0.69, 0.95)
Gly	6.02<br>(1.42, 10.62)	4.79<br>(4.19, 5.39)	2.79<br>(2.19, 3.39)	5.09<br>(4.0, 6.17)	3.63<br>(1.18, 6.08)	2.96<br>(1.54, 4.38)	7.34<br>(0.36, 14.33)	5.42<br>(3.11, 7.73)	4.42<br>(3.85, 4.99)
Thr	0.66<br>(0.57, 0.75)	1.44<br>(0.6, 2.28)	-0.12<br>(-0.96, 0.73)	0.61<br>(0.03, 1.18)	1.14<br>(0.47, 1.81)	0.05<br>(-0.41, 0.51)	0.89<br>(-1.1, 2.87)	1.78<br>(0.87, 2.68)	0.1<br>(-1.84, 2.04)
Cit	0.22<br>(0.17, 0.27)	0.29<br>(0.25, 0.32)	0.03<br>(-0.01, 0.07)	0.15<br>(0.11, 0.21)	0.13<br>(0.08, 0.21)	0.23<br>(0.15, 0.32)	0.26<br>(0.14, 0.38)	0.31<br>(0.13, 0.52)	0.17<br>(0.09, 0.26)
Arg	-1.01<br>(-1.35, -0.65)	-0.08<br>(-0.25, 0.09)	-2.41<br>(-2.58, -2.23)	-0.82<br>(-1.02, -0.62)	-0.46<br>(-0.91, -0.01)	-1.26<br>(-2.04, -0.48)	-0.17<br>(-0.97, 0.64)	0.11<br>(-0.85, 1.06)	-1.49<br>(-3.53, 0.55)
Ala	9.35<br>(5.58, 13.13)	9.74<br>(7.81, 11.74)	16.73<br>(14.78, 18.68)	3.52<br>(3.35, 3.68)	4.44<br>(3.05, 6.26)	7.88<br>(6.41, 9.35)	7.74<br>(0.91, 14.58)	11.04<br>(1.33, 20.76)	7.12<br>(4.41, 9.83)
Tyr	0.82<br>(0.78, 0.85)	0.77<br>(0.60, 0.93)	0.46<br>(0.29, 0.62)	0.57<br>(0.12, 1.03)	0.52<br>(0.17, 0.86)	1.11<br>(0.84, 1.38)	0.93<br>(-0.54, 2.4)	1.45<br>(0.88, 2.02)	-0.08<br>(-1.38, 1.23)
Val	2.51<br>(1.75, 3.26)	1.05<br>(1.03, 1.06)	0.40<br>(0.39, 0.41)	0.18<br>(-0.43, 0.79)	1.16<br>(0.15, 2.16)	1.10<br>(0.95, 1.24)	0.43<br>(0.34, 0.51)	1.66<br>(1.42, 1.89)	-0.38<br>(-1.53, 0.78)
Met	0.38<br>(0.11, 0.65)	0.33<br>(0.13, 0.53)	-0.03<br>(-0.23, 0.17)	0.03<br>(-0.14, 0.21)	0.35<br>(0.07, 0.64)	0.43<br>(0.19, 0.66)	0.14<br>(-0.66, 0.94)	0.54<br>(0.50, 0.57)	-0.37<br>(-0.87, 0.14)
Trp	0.05<br>(0.01, 0.08)	0.16<br>(0.11, 0.21)	-1.37<br>(-1.42, -1.32)	0.07<br>(0.00, 0.13)	0.18<br>(0.03, 0.33)	-0.14<br>(-0.22, -0.05)	0.03<br>(-0.17, 0.23)	0.29<br>(0.17, 0.41)	-0.21<br>(-0.24, -0.17)
Phe	1.11<br>(1.09, 1.12)	0.89<br>(0.62, 1.15)	0.51<br>(0.25, 0.77)	0.25<br>(-0.06, 0.56)	0.71<br>(0.01, 1.4)	0.75<br>(0.46, 1.03)	0.25<br>(-0.18, 1.22)	1.1 1<br>(-0.39, 2.00)	-0.44<br>(-0.77, -0.10)
Orn	1.51<br>(1.09, 1.79)	0.42<br>(-0.45, 1.29)	-0.58<br>(-1.42, 0.32)	-0.66<br>(-0.99, -0.32)	-0.72<br>(-0.75, -0.68)	-0.82<br>(-1.03, -0.62)	1.00<br>(-0.24, 2.25)	1.43<br>(1.11, 1.74)	0.04<br>(-1.70, 1.78)
Leu	0.11<br>(-0.66, 0.88)	1.23<br>(-0.09, 2.55)	-1.18<br>(-2.5, 0.14)	0.42<br>(0.23, 0.64)	0.22<br>(-0.21, 0.62)	1.91<br>(0.92, 2.90)	0.64<br>(-1.99, 3.28)	2.57<br>(2.53, 2.64)	-1.22<br>(-3.98, 1.54)
Lys	1.5<br>(-1.13, 4.12)	-0.33<br>(-1.27, 0.61)	-1.85<br>(-2.8, -0.9)	0.88<br>(-1.05, 2.81)	-0.49<br>(-1.32, 0.35)	-0.92<br>(-1.22, -0.62)	1.16<br>(-2.12, 4.44)	2.21<br>(1.04, 3.38)	-1.67<br>(-2.2, -1.13)
Ile	2.06<br>(1.31, 2.81)	-0.66<br>(-0.77, -0.54)	-1.83<br>(-1.94, -1.72)	0.91<br>(0.62, 1.19)	0.91<br>(0.59, 1.25)	-0.45<br>(-1.34, 0.53)	0.32<br>(-1.47, 2.06)	1.47<br>(1.07, 1.87)	-1.11<br>(-2.91, 0.7)

Supplementary Table S2. Effect of urea supplementation (0, 20, or 40 mg/dl) in maturation medium on the net depletion/appearance of amino acids by bovine cumulus-oocyte complex (COCs), oocytectomized cumulus cells (CCs), or denuded oocytes (DOs) after 24-h incubation. The means and 95% confidence intervals derived from three samples (three observations per replicate). The 95% confidence interval covered the mean in all observations. The sample size was n = 3. The degree of freedom (df) = n-1 =2. The t value for 95% confidence with df = 2 was t = 4.303. U0: control (without urea); U20: 20 mg/dl urea; U40: 40 mg/dl urea.

Amino acid	Reference value, %	Detected values, %				Recovery, %
		1	2	3	Average	
**Asp**	0.915	0.952	1.112	1.021	1.028	112.4
**Glu**	1.231	1.232	1.178	1.120	1.177	95.6
**Ser**	0.752	0.751	0.695	0.688	0.711	94.6
**Gln**	0.912	0.943	0.932	0.996	0.957	104.9
**His**	0.652	0.644	0.639	0.649	0.644	98.8
**Gly**	0.458	0.462	0.482	0.492	0.479	104.5
**Thr**	0.625	0.627	0.725	0.629	0.660	105.7
**Arg**	0.963	1.023	1.052	0.962	1.012	105.1
**Ala**	0.748	0.824	0.789	0.752	0.788	105.4
**Tyr**	0.385	0.325	0.384	0.381	0.363	94.4
**Val**	0.689	0.698	0.742	0.741	0.727	105.5
**Met**	0.414	0.419	0.411	0.382	0.404	97.6
**Trp**	0.584	0.562	0.524	0.545	0.544	93.1
**Phe**	0.632	0.689	0.684	0.635	0.669	105.9
**Leu**	1.583	1.635	1.589	1.852	1.692	106.9
**Lys**	0.687	0.756	0.745	0.698	0.733	106.7
**Ile**	0.665	0.741	0.71	0.672	0.708	106.4

Supplementary Table S3. Percentage of recovery for each amino acid in the reference medium. The measurement was repeated three times using HPLC.

Amino acid	Amino acid concentrations (μmol/ml)					*RSD*, %
	1	2	3	4	Average	
**Asp**	0.151	0.149	0.155	0.161	0.154	3.44
**Glu**	0.401	0.391	0.402	0.422	0.404	3.21
**Ser**	0.171	0.182	0.175	0.186	0.179	3.79
**Gln**	0.388	0.398	.401	0.419	0.402	3.22
**His**	0.093	0.094	0.089	0.097	0.093	3.54
**Gly**	0.615	0.611	0.602	0.647	0.619	3.17
**Thr**	0.169	0.180	0.172	0.184	0.176	3.94
**Arg**	0.262	0.261	0.271	0.280	0.269	3.31
**Cit**	0.018	0.019	0.019	0.020	0.019	4.30
**Ala**	0.316	0.315	0.300	0.329	0.315	3.77
**Tyr**	0.171	0.171	0.176	0.183	0.175	3.24
**Val**	0.160	0.166	0.162	0.173	0.165	3.47
**Met**	0.080	0.082	0.081	0.086	0.082	3.20
**Trp**	0.046	0.044	0.043	0.047	0.045	4.06
**Phe**	0.122	0.120	0.118	0.127	0.122	3.17
**Orn**	0.018	0.019	0.019	0.020	0.019	4.30
**Leu**	0.350	0.351	0.369	0.378	0.362	3.81
**Lys**	0.328	0.331	0.348	0.356	0.341	3.95
**Ile**	0.232	0.230	0.222	0.242	0.232	3.55

Supplementary Table S4. Repeatability of amino acids determination in the fresh maturation medium using HPLC method. The obtained *RSD* values were in the acceptable range of 1.12 to 3.36%.
